# PW01-026 – Validation of pediatric diagnostic criteria in FMF

**DOI:** 10.1186/1546-0096-11-S1-A79

**Published:** 2013-11-08

**Authors:** E Demirkaya, S Ozen, C Saglam, T Turker, A Duzova, P Woo, I Konè-Paut, M Doglio, G Amaryan, J Frenkel, Y Uziel, A Insalaco, L Cantarini, M Hofer, S Boiu, C Modesto, A Bryant, D Rigante, E Papadopoulou-Alataki, S Guillaume-Czitrom, N Ruperto, M Gattorno

**Affiliations:** 1 FMF Arthritis Vasculitis and Orphan Disease Research Center (FAVOR), Gulhane Military Medical Faculty, Ankara, Turkey; 2 Pediatric Rheumatology, Hacettepe University, School of Medicine, Ankara, Turkey; 3 Epidemiology, Gulhane Military Medical Faculty, Ankara, Turkey; 4 Rheumatology, Istanbul University, Istanbul, Turkey; 5 Pediatric Rheumatology, UCL, London, UK; 6 Pediatric Rheumatology, University of Paris SUD, Paris, France; 7 Pediatric Rheumatology, Ospedale Gaslini, Genoa, Italy; 8 National Pediatric Familial Mediterranean Fever Centre, Institute of Child and Adolescent Health, Yerevan, Armenia; 9 Pediatrics, University Medical Center Utrecht, Utrecht, the Netherlands; 10 Pediatrics, Meir Medical Centre, Kfar Saba, Israel; 11 Reumatologia, Ospedale Pediatrico Bambin Gesù, Rome, Italy; 12 Rheumatology, Policlinico le Scotte, University of Siena, Siena, Italy; 13 Pediatric Rheumatology, Centre Hospitalier Universitaire Vaudois, Lausanne, Switzerland; 14 Pediatric Rheumatology, Université Paris-Descartes, Paris, France; 15 Reumatologia, Hospital Valle de Hebron, Barcelona, Spain; 16 Pediatrics, Università Cattolica Sacro Cuore, Rome, Italy; 17 Fourth Department of Pediatrics, Aristotle University of Thessaloniki Papageorgiou Hospital, Thessaloniki, Greece; 18 Pediatric Rheumatology, Bicêtre-Hôpitaux Universitaires Paris-Sud, Bicetre, France

## Introduction

The diagnosis of FMF is made clinically and may be confirmed by identifying mutations in the *MEFV* gene. The most commonly used diagnostic criteria for FMF are those of Tel Hashomer, which have been established in the Jewish adult population. Recently, a Turkish group (Yalcinkaya-Ozen's diagnostic criteria) proposed new criteria for diagnosis of FMF in children.

## Objectives

We analyzed the validity and reliability of Yalcinkaya-Ozen’s diagnostic criteria for childhood FMF in a large international registry.

## Methods

The study group consisted of 339 FMF patients diagnosed according to Tel Hashomer criteria. A control group of 377 patients were diagnosed other periodic fever syndromes including MKD, TRAPS, CAPS and PFAPA syndromes. Both groups were evaluated according to the Tel Hashomer criteria and the new set of diagnostic criteria proposed to use in childhood FMF. The diagnostic performance of both criteria was assessed by multiple logistic regression analysis.

## Results

The sensitivity and specificity of Tel Hashomer criteria in our study were 35.1% and 97.7 %, respectively. The presence of two or more of these new five criteria diagnosed FMF with a high and sufficient sensitivity of 87.4 % and the NPV of 74.8%. When we used at least three Yalcinkaya-Ozen’s criteria, the discrimination of the diseases other than FMF reached the highest specificity of 88.2% and the PPV of 82.9% however the sensitivity was compromised. In case of all the new set of criteria were met, the sensitivity and specificity were 99.6% and 5.6%, respectively with a PPV of 94.1% and an NPV of 49.2% . Our study showed that ethnicity had no impact on the validation.

## Conclusion

Tel Hashomer diagnostic criteria was found to have high specificity, whereas Yalçınkaya-Ozen’s criteria has a higher sensitivity for the diagnosis of FMF with the combination of at least any two out of these five criteria are met. The small number of patients with amyloidosis or erysipelas like erythema and the response to colchicine therapy constituted the drawbacks in assessing the patients with Tel Hashomer criteria.

## Disclosure of interest

None declared

